# Supplementary material for: Grb7, Grb10 and Grb14, encoding the growth factor receptor-bound 7 family of signalling adaptor proteins have overlapping functions in the regulation of fetal growth and post-natal glucose metabolism
Source: BMC Biol. 2024 Sep 30;22:221. doi: 10.1186/s12915-024-02018-5 (PMC11441139; doi:10.1186/s12915-024-02018-5)
Supplement: Supplementary file 1 — Additional file 1: Figures S1-S4. Fig. S1. Comparison of expression patterns during fetal development for Grb7, Grb10 and Grb14 gene. (A-C) Protein expression in a wild type e14.5 embryo, determined by antibody staining for Grb7, Grb10, and Grb14. The images for Grb7 (A) and Grb14 (C) are the same as those shown in Figs. 1 and 2, respectively, and the image for Grb10 (B) is from our earlier publication [17]. Expression is highlighted in various tissues: cardiac muscle (c), choroid plexus (cp), dermis (d), diaphragm (di), gut (g), inner ear (i), kidney (k), liver (li), lung (lu), mid brain (mb), nasal epithelium (ne), pancreas (p), pituitary (pi), ribs (r), stomach (s), salivary gland (sg), skeletal muscle (sk), tongue (t), and tooth primordia (tp). (D-F) Messenger RNA expression at daily intervals from e9.5 to e16.5 taken from the MOSTA spatial transcriptomics database (https://db.cngb.org/stomics/mosta/spatial/) [48]. Relative expression levels are indicated by the colour intensity, based on the number of transcript reads for Grb7 (D), Grb10 (E) and Grb14 (F). Fig. S2. Southern blot analysis of HindIII digested DNA from wild type ES cells (ES) or primary mouse embryonic fibroblasts (MEF) alongside three successfully targeted ES cell clones (2E5, 4B5 and 5D1). Images are of the full-length blots shown in truncated form in Fig. 5B. In the targeted (TG) allele, loss of the sequence between the homologous arms alters the distance between restriction enzyme sites, compared with the wild type (WT) allele. Consequently, in the wild type allele Probe A recognises a 5′ 8.7 kb fragment and Probe B a 3′ 10.8 kb fragment, whereas both probes detect a 15.3 kb fragment for the targeted allele. Fig. S3. Organ and tissue weight data for adult Grb7 KO mice. Additional tissue and organ weights were obtained for the same animals described in Fig. 8. Raw weights are shown for both males (A- G) and females (O-T), alongside the weight of each tissue as a proportion of body weight for ma [file 12915_2024_2018_MOESM1_ESM.pptx]

## Slide 1
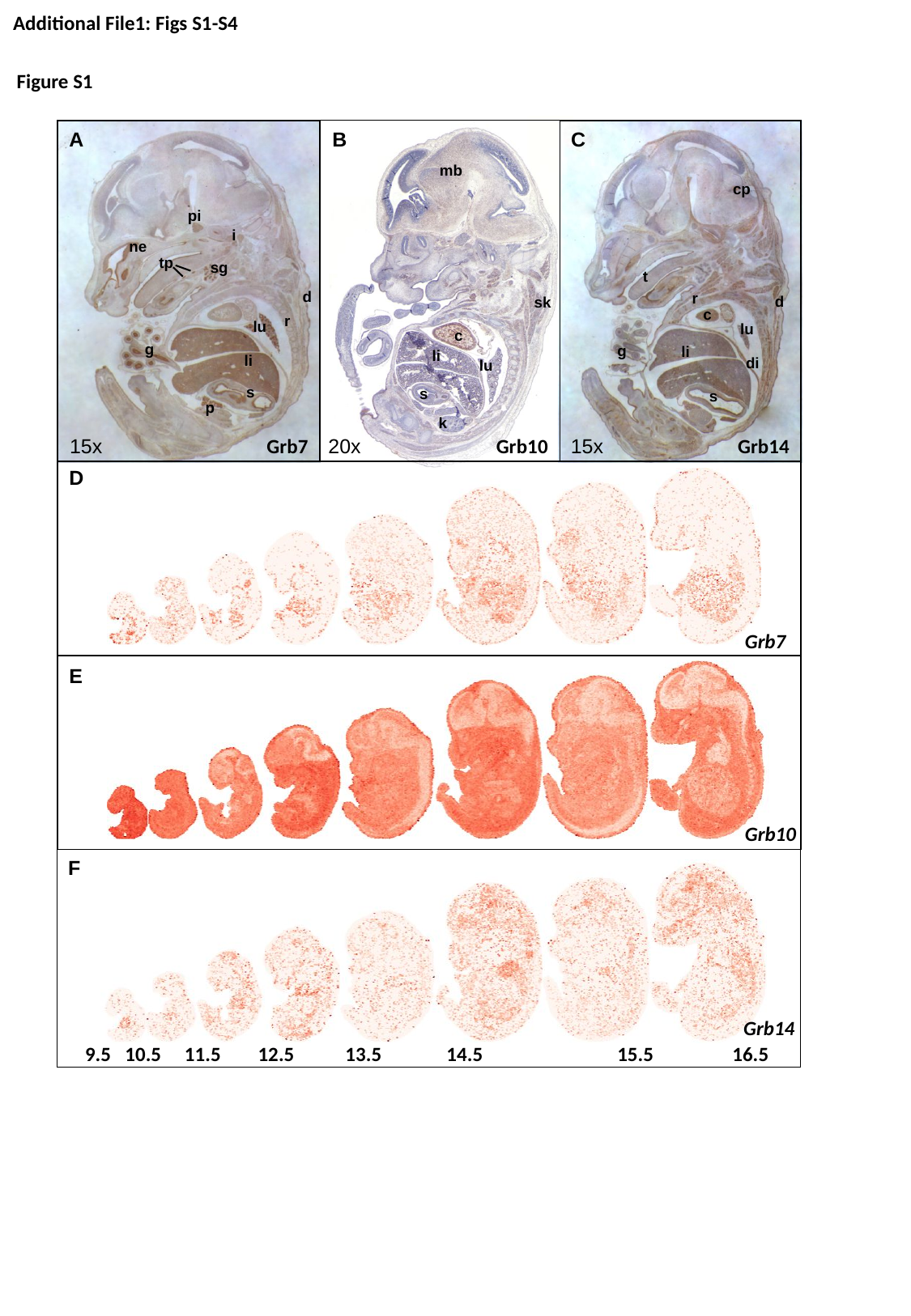

Additional File1: Figs S1-S4
Figure S1
A
B
C
mb
cp
pi
i
ne
tp
sg
t
d
r
d
sk
c
r
lu
lu
c
g
g
li
li
li
di
lu
s
s
s
p
k
15x
15x
Grb7
20x
Grb10
Grb14
D
Grb7
E
Grb10
F
Grb14
9.5 10.5 11.5 12.5 13.5 14.5	 15.5 16.5

## Slide 2
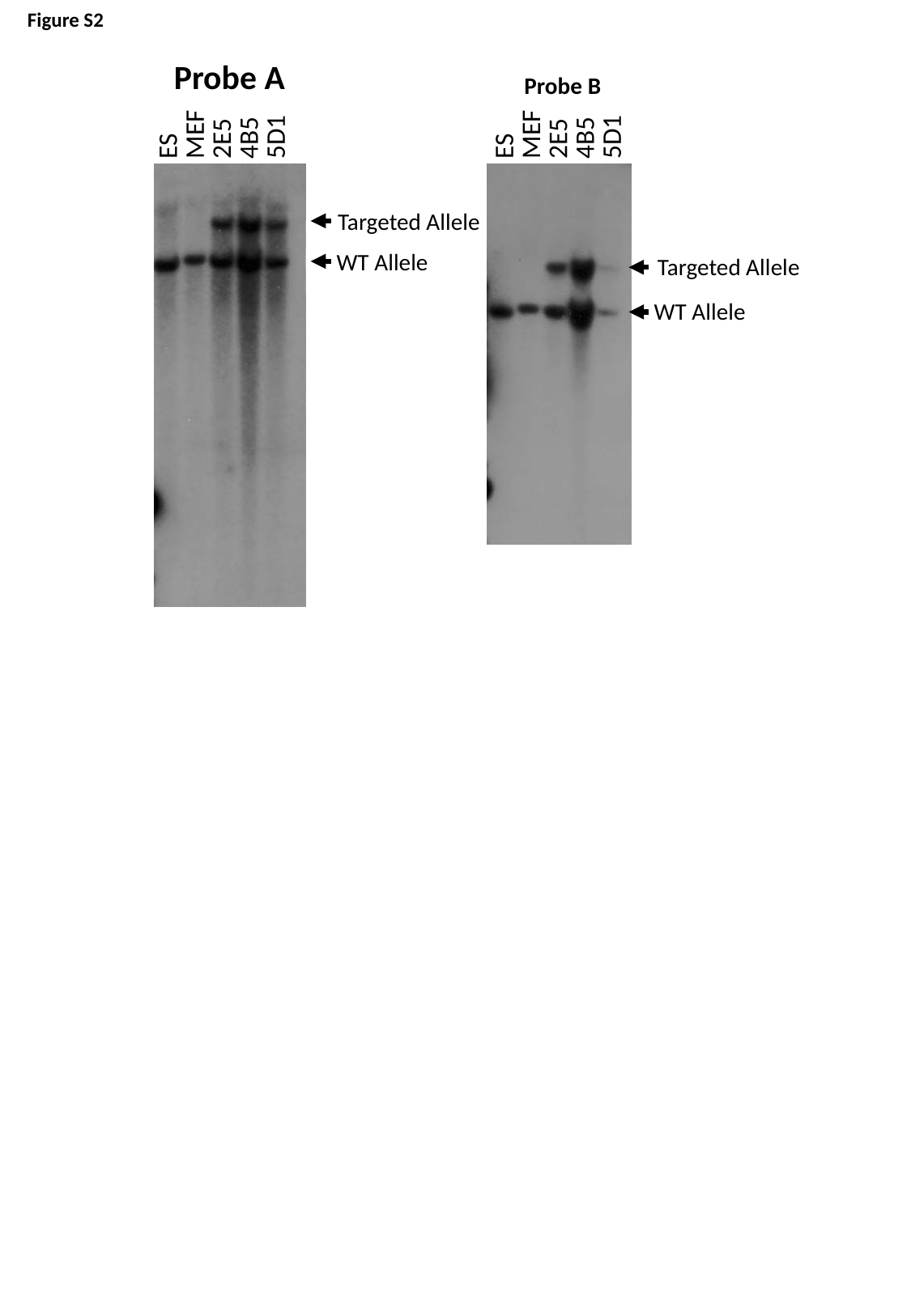

Figure S2
ES
MEF
2E5
4B5
5D1
ES
MEF
2E5
4B5
5D1
Probe A
Probe B
Targeted Allele
WT Allele
Targeted Allele
WT Allele

## Slide 3
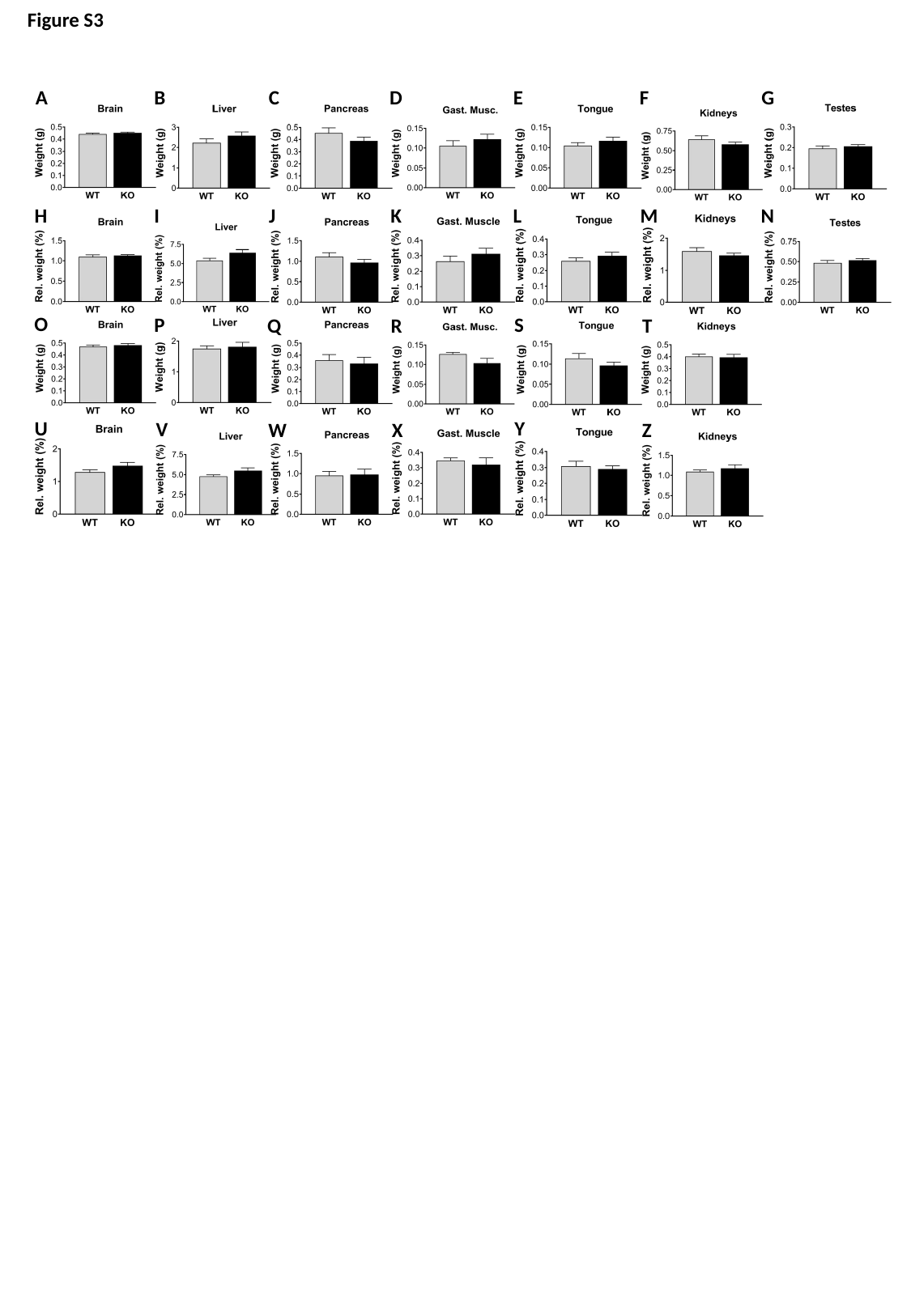

Figure S3
A
B
C
D
E
F
G
l
H
I
J
K
L
M
N
O
P
S
Q
R
T
Y
U
V
Z
W
X

## Slide 4
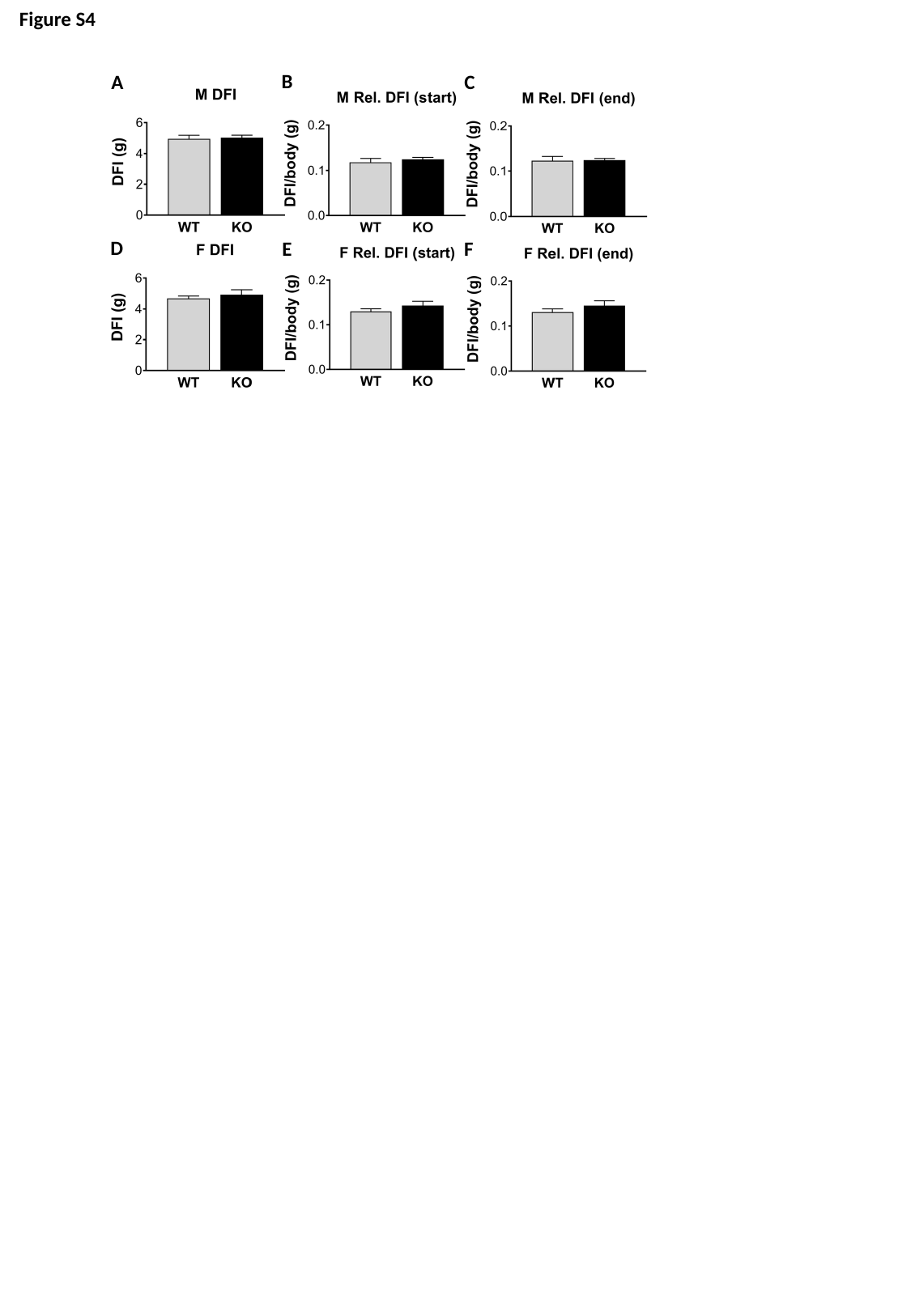

Figure S4
B
A
C
D
F
E
