# Supplementary material for: Grb7, Grb10 and Grb14, encoding the growth factor receptor-bound 7 family of signalling adaptor proteins have overlapping functions in the regulation of fetal growth and post-natal glucose metabolism
Source: BMC Biol. 2024 Sep 30;22:221. doi: 10.1186/s12915-024-02018-5 (PMC11441139; doi:10.1186/s12915-024-02018-5)
Supplement: Supplementary file 2 — Additional file 2. [file 12915_2024_2018_MOESM2_ESM.docx]

**Additional file 2: Tables S1-S2**

**Table S1.**

| **Chi-square test** |  |
| --- | --- |
| Chi-square | 6.154 |
| DF | 11 |
| P value (two-tailed) | 0.8629 |
| P value summary | ns |
| Is discrepancy significant (P < 0.05)? | No |

| **Outcome** | **Expected #** | **Observed #** | **Expected %** | **Observed %** |
| --- | --- | --- | --- | --- |
| 10WT:14WT | 3.25 | 3 | 6.25 | 5.769 |
| 10WT:14HET | 6.5 | 10 | 12.5 | 19.23 |
| 10WT:14KO | 3.25 | 4 | 6.25 | 7.692 |
| 10P:14WT | 3.25 | 3 | 6.25 | 5.769 |
| 10P:14HET | 6.5 | 9 | 12.5 | 17.31 |
| 10P:14KO | 3.25 | 3 | 6.25 | 5.769 |
| 10M:14WT | 3.25 | 1 | 6.25 | 1.923 |
| 10M:14HET | 6.5 | 4 | 12.5 | 7.692 |
| 10M:14KO | 3.25 | 3 | 6.25 | 5.769 |
| 10KO:14WT | 3.25 | 3 | 6.25 | 5.769 |
| 10KO:14HET | 6.5 | 5 | 12.5 | 9.615 |
| 10KO:14KO | 3.25 | 4 | 6.25 | 7.692 |
| TOTAL | 52 | 52 | 100 | 100 |

**Table S2.**

**A)** Chi-square test: *Grb10^+/p^:Grb7^+/-^* female x *Grb10^+/+^:Grb7^+/-^* male

PN1 offpsring

| **Chi-square test** |  |
| --- | --- |
| Chi-square | 5.433 |
| DF | 5 |
| P value (two-tailed) | 0.3653 |
| P value summary | ns |
| Is discrepancy significant (P < 0.05)? | No |

| **Outcome** | **Expected #** | **Observed #** | **Expected %** | **Observed %** |
| --- | --- | --- | --- | --- |
| 10WT:7WT | 15 | 10 | 12.5 | 8.33 |
| 10WT:7HET | 30 | 32 | 25 | 26.67 |
| 10WT:7KO | 15 | 14 | 12.5 | 11.67 |
| 10KO:7WT | 15 | 22 | 12.5 | 18.33 |
| 10KO:7HET | 30 | 27 | 25 | 22.50 |
| 10KO:7KO | 15 | 15 | 12.5 | 12.50 |
| TOTAL | 120 | 120 | 100 | 100 |

**B)** Chi-square test: *Grb10^+/p^:Grb7^+/-^* female x *Grb10^+/+^:Grb7^+/-^* male

e17.5 offspring

| **Chi-square test** |  |
| --- | --- |
| Chi-square | 2.803 |
| DF | 5 |
| P value (two-tailed) | 0.7303 |
| P value summary | ns |
| Is discrepancy significant (P < 0.05)? | No |

| **Outcome** | **Expected #** | **Observed #** | **Expected %** | **Observed %** |
| --- | --- | --- | --- | --- |
| 10WT:7WT | 7.625 | 8 | 12.5 | 13.11 |
| 10WT:7HET | 15.25 | 15 | 25 | 24.59 |
| 10WT:7KO | 7.625 | 5 | 12.5 | 8.197 |
| 10KO:7WT | 7.625 | 11 | 12.5 | 18.03 |
| 10KO:7HET | 15.25 | 16 | 25 | 26.23 |
| 10KO:7KO | 7.625 | 6 | 12.5 | 9.84 |
| TOTAL | 61 | 61 | 100 | 100 |
